# Intrathecal Immunoglobulin A Synthesis in Multiple Sclerosis: From Biological Aspects to Clinical Relevance

**DOI:** 10.3390/biom15010108

**Published:** 2025-01-11

**Authors:** Dariia Kliushnikova, Ferdinand Otto, Georg Pilz, Peter Wipfler, Andrea Harrer

**Affiliations:** 1 Department of Neurology, Christian-Doppler University Hospital, Paracelsus Medical University, 5020 Salzburg, Austria; f.otto@salk.at (F.O.); georg.pilz@salk.at (G.P.); p.wipfler@salk.at (P.W.); 2 Department of Neurology, University Hospital Zurich, University of Zurich, 8006 Zürich, Switzerland; 3 Department of Dermatology and Allergology, Paracelsus Medical University, 5020 Salzburg, Austria

**Keywords:** IgA, multiple sclerosis, cerebrospinal fluid, prognosis, intrathecal immunoglobulin synthesis

## Abstract

Intrathecal immunoglobulin A (IgA) synthesis in multiple sclerosis (MS) has long earned little attention, despite a potential significance in disease pathogenesis and prognosis. The presence of IgA-positive plasma cells in MS lesions and along damaged axons suggests a role in disease pathogenesis. Available clinical evidence about a potential positive or negative prognostic role is scarce and inconclusive. Recent observations, however, highlight the migration of immune regulatory IgA-producing plasma cells from the gut to the central nervous system (CNS) in experimental autoimmune encephalitis models. A connection between intrathecal IgA synthesis and the gut–brain axis in MS was further corroborated by the discovery of gut microbiota-specific IgA+ B cells in human CNS during relapse. In this review, we summarize current evidence on the occurrence and immunopathology of intrathecal IgA synthesis in MS, explore its biological implications, and address methodological challenges regarding the detection of IgA as a major limitation and possible source of inconsistencies in clinical studies. By synthesizing these diverse lines of evidence, we highlight the importance of further research and the need for standardized detection methods to clarify the role of IgA in MS pathogenesis, disease progression, and as potential biomarker.

## 1. Introduction

Intrathecal immunoglobulin (Ig) synthesis is a hallmark of humoral B cell-mediated immune activity observed in various neuroinflammatory conditions [1]. While indicating an adaptive humoral immune response against bacterial or viral pathogens, it is also implicated in immune-mediated neuroinflammation, particularly multiple sclerosis (MS) [2,3].

MS is a chronic inflammatory demyelinating disease of the central nervous system (CNS) with B cells significantly contributing to its pathogenesis, as evidenced by the effectiveness of B cell-depleting therapies [4,5,6]. However, these therapies do not target plasma cells, the antibody-producing effector cells, leaving the specific functions of B cells and their progeny in MS pathology unclear [7].

Intrathecal IgG synthesis, detected through quantitative methods like the IgG index and Reiber’s nephelometry [8,9] or qualitatively through isoelectric focusing for oligoclonal bands (OCBs), has been a diagnostic hallmark of MS for more than half a century [10]. Cerebrospinal fluid (CSF)-specific OCB is the most consistent immunological biomarker to diagnose MS and is present in the CSF in 90–95% of patients with MS. Though intrathecal IgG has been associated with earlier conversion to definitive MS [11] and disability worsening in MS patients [12], the immunopathological role of intrathecal IgG remains elusive. Similarly, the significance of intrathecal IgM, associated with a more aggressive disease course, is not fully understood [13,14,15,16].

Even more enigmatic is the role of intrathecal IgA, found in approximately 9–18% of MS patients [17]. The limited existing studies on intrathecal IgA in MS have reported conflicting findings regarding its effects on disease prognosis. This knowledge gap requires attention, as the presence of intrathecal IgA in neuroinflammation, coupled with recent insights into the gut–brain axis, suggests a role for IgA beyond mucosal immunity [18].

This review synthesizes the current evidence on the occurrence and immunopathology of intrathecal IgA synthesis in MS. We also discuss the methodological aspects of its detection. Given that intrathecal IgA synthesis implies the presence of IgA+ B cells and IgA-secreting plasma cells, we begin with a concise overview of general B cell biology and IgA-associated humoral immunity.

## 2. IgA-Associated B Cell Biology

B cells originate from hematopoietic stem cells and mature in the bone marrow before they enter the circulation as mature naive B cells, passing secondary lymphoid organs (SLO) like the spleen, lymph nodes, and mucosal-associated lymphoid tissues (MALTs). In the case of antigen stimulation in these SLO, naive B cells differentiate into specialized subtypes such as plasmablasts, long-lived plasma cells, short-lived plasma cells, and memory B cells [19]. Antibody responses can be T cell-dependent and T cell-independent, relying on the nature of the antigen and the involvement of helper T cells. T cell-independent antibody responses are formed by short-lived plasma cells, which produce mainly low-affinity IgM antibodies. T cell-dependent antibody responses result in the formation of germinal centers in SLO and are characterized by somatic hypermutation, antibody affinity maturation, immunoglobulin class switching, and differentiation to memory B cells and plasma cells. Long-lived plasma cells produce high-affinity antibodies of either the IgG, IgA, or IgE class, and usually home to the bone marrow [20]. There, they maintain the ability to produce antibodies without requiring re-stimulation by antigen, which is the most critical feature of humoral adaptive immune protection by immediately and selectively combating pathogens as antibody-based immunological memory [21].

**Figure 1:**
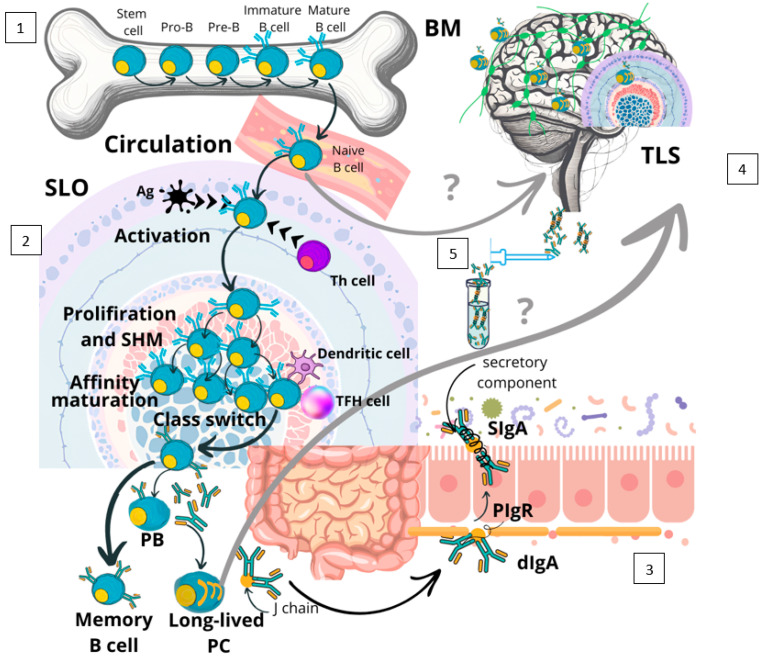
The immune biology of IgA in MALTs and potential connection to the CNS. Naive B cells enter the circulation after maturation in the bone marrow and migrate to secondary lymphoid structures in the mucosa-associated lymphoid tissue (1). Once activated by antigen, they can interact with cognate T cells and thereby initiate T cell-dependent antibody responses via the formation of germinal centers in SLO. This process is characterized by somatic hypermutation, antibody affinity maturation, immunoglobulin class switching, and differentiation into IgA+ memory B cells and IgA-secreting plasmablasts/cells (2). Mucosa-associated plasma blasts/cells exit the SLO via the lymph, enter the circulation, home back into their mucosal tissue via special homing receptors (e.g., Alpha4beta7 integrin and MADCAM1), and secrete dimeric IgA. The dimeric IgA binds to polymeric immunoglobulin receptors on epithelial cells, which during transcytosis are stabilized by the secretory component and released to mucosal surfaces as secretory IgA (3). Long-lived IgA+-PC physiologically reside in MALTs but can also be found in the CNS, for instance, in meningeal TLSs (4). Whether IgA+ plasma cells/plasma blasts or mature naive IgA+ B cells migrate from the gut into the inflamed CNS is not definitively known; both pathways are suspected. IgA can be detected in CSF through lumbar puncture (5). Abbreviations: BM, bone marrow; dIgA, dimeric immunoglobulin A; PB, plasma blast; PC, plasma cell; PIgR, polymeric immunoglobulin receptor; SIgA, secretory immunoglobulin A; SLO, secondary lymphoid organ; TFH cell, T follicular helper cell; Th cell, T helper cell; and TLS, tertiary lymphoid structure.

IgA+ plasma cells mainly home to gut-associated lymphoid tissue (GALT, which includes Peyer’s patches, the appendix, and scattered solitary or isolated lymphoid follicles), where they produce monomeric, dimeric, and sometimes polymeric IgA, with the two latter linked by the J chain. Dimeric but also polymeric IgA can bind to the polymeric immunoglobulin receptor on epithelial cells, whereupon internalization they are further equipped and stabilized by the so-called secretory component and released to mucosal surfaces as secretory IgA [22]. In the peripheral blood, IgA is the second most abundant immunoglobulin class, comprises around 15% of total peripheral blood immunoglobulins, and predominantly exists in the monomeric form [23]. Whereas IgM and IgG are primarily found in the circulatory system and SLO, IgA is the predominant immunoglobulin in the MALT system, which is the respiratory, gastrointestinal, and genitourinary tract [24]. There, IgA neutralizes pathogens, maintains epithelial barrier function, and regulates the commensal microbiome [18,24,25] (Figure 1). The two main IgA subclasses, IgA1 and IgA2, exhibit distinct glycosylation patterns and tissue distribution. IgA1 accounts for 80–85% of total serum IgA and is prevalent at various mucosal sites, including the nasal, bronchial, gastric, and small intestinal mucosa. In contrast, IgA2 is primarily found in the colon and is more resistant to bacterial proteases [26,27]. Interestingly, these IgA subclasses possess divergent effector functions, as IgA2 has been shown to exert pro-inflammatory effects on neutrophils and macrophages, while IgA1 is primarily associated with immune homeostasis [28].

IgA maintains epithelial barrier function mainly by a mechanism called immune exclusion, which is accomplished by trapping antigens for removal, hence limiting bacterial colonization and avoiding potential immune responses [29]. Intestinal homeostasis is accomplished by shaping the composition of the commensal gut microbiota [29,30]. This functional diversity also suggests a role of IgA in oral tolerance, which is a form of peripheral tolerance that produces a state of immune unresponsiveness to harmless external antigens. Oral tolerance develops as a result of a complex interplay between innate and adaptive immune functions and known mechanisms involving anergy, clonal deletion, or active induction of regulatory T cells [29,31]. Notably, oral antigen administration elicits local secretion of non-inflammatory IgA in the gut mucosa and its draining lymph nodes, and although oral tolerance is established in the gut, it apparently can spread out to the entire body leading to systemic tolerance [31]. How systemic and mucosal IgA responses contribute to the development of oral tolerance is still unknown but is an area of intensive research.

## 3. The Connection Between IgA, CNS Immunity, and MS

The brain was long considered an “immune-privileged” organ, shielded from the peripheral immune system by specialized barriers, such as the blood–brain barrier, the blood–cerebrospinal fluid barrier, the blood–leptomeningeal barrier, and the blood–retinal barrier [32,33]. Depending on the context and cause of CNS inflammation, these barriers may become permeable, thereby enabling the infiltration of peripheral blood immune cells, including B cells, T cells, and plasmablasts/cells into the CSF compartment of the CNS and/or brain parenchyma [34]. This infiltration can result in the formation of niches that sustain the survival of non-proliferating, potentially long-lived plasma cells at parenchymal, perivascular, and meningeal locations, observed in brain biopsies of MS patients [35]. Recent work further highlighted a local B cell maturation into antibody-secreting plasma cells/plasmablasts [36]. Preferentially observed in active lesions, the local transition of B cells was linked to intrathecal immunoglobulin production and likely depended on interaction with CD4+ memory T cells [36].

Besides brain parenchymal lesions and perivascular spaces, tertiary lymphoid structures (TLSs) in the inflamed meninges may also serve as niches for compartmentalized B cell responses and accumulation of plasmablasts/plasma cells [37]. TLSs are highly organized follicle-like structures that can emerge at sites of chronic tissue inflammation outside of SLO. The cellular organization within TLS-like structures detected in brain tissues is heterogeneous, ranging from sites of simple B cell accumulations to complex structures containing B cells, T cells, follicular dendritic cells, and plasma cells/blasts [38,39]. TLS-like structures have been detected in up to 40–60% of post-mortem tissue from individuals with MS, preferentially located in the meninges of the cerebral hemispheres, cerebellum, brain stem, and spinal cord [40]. Their presence is often associated with adjacent cortical demyelination and a more severe disease course with younger age at disease onset, younger age at irreversible disability, and earlier death [40,41,42].

IgA-producing cells have been observed infiltrating the brains of MS patients [43]. Immunocytochemistry of demyelinating plaques has revealed numerous IgA-producing plasma cells present in perivascular spaces and brain parenchyma, correlating with axon loss, which points to the involvement of IgA+ plasma cells in MS pathogenesis [44]. Conversely, Rodriguez-Mogeda failed to quantify IgA-producing cells in post-mortem brain samples from 20 MS patients, suggesting that IgA may not play a significant role in the pathogenesis of the disease [45]. IgA+ B cells otherwise have been reported to occur in inflammatory meningeal aggregates and in parenchymal and perivascular spaces of the inflamed subcortical white matter, though to a significantly lesser extent compared to IgG+ B cells [46]. Notably, the same study observed IgA+ B and plasma cells in the inflamed CSF, which were clonally related to those in the peripheral blood and intrathecal IgA production during MS relapse, suggesting active exchange during active inflammation in MS relapse [46].

All of the above-mentioned studies support a yet underestimated role for IgA, IgA+ B cells, and IgA+ plasma cells/plasmablasts in CNS inflammation during MS and call for a re-evaluation of the current knowledge. Normal CSF contains mainly monomeric IgA. However, during neuroinflammation, the dimeric form of IgA becomes more prevalent. This was clearly demonstrated by Sindic et al. in their study conducted 40 years ago by investigating dimeric intrathecal IgA production in the CSF of patients with various neuroinflammatory conditions, including MS, tuberculous meningitis, and herpetic encephalitis [47]. The authors applied ultracentrifugation to differentiate monomeric form dimeric IgA in both blood and CSF and determined intrathecal IgA production for both IgA forms separately. Although in controls the proportion of dimeric IgA was less than 5% of total IgA, it increased to more than 50% in cases with intrathecal IgA production in the inflamed CSF. They also conducted IgA1 vs. IgA2 subtyping in nine patients, including three with MS. Their findings revealed that IgA1 was the predominant subclass in these patients. The predominance of IgA1 in their cohort supports the hypothesis that viral antigens may play a role in MS pathogenesis, as the IgA1 subclass is preferentially induced in response to viral stimuli [48].

Intrathecal IgA production hence is primarily of the IgA1 subclass and is based on the presence of intrathecal dimeric IgA. Importantly, one MS patient with a normal total IgA index had a markedly elevated index for dimeric IgA, suggesting that intrathecal dimeric IgA responses may be masked by normal total IgA values. Dimeric IgA apparently plays a different role in neuroinflammatory processes than monomeric IgA, about which we have no knowledge, and may even underestimate its presence because routinely used nephelometry cannot differentiate between these two IgA forms [47].

### 3.1. The Role of Intrathecal Dimeric IgA

So what could be the role of intrathecally produced dimeric IgA in neuroinflammation and can we draw any conclusion from the periphery? In contrast to mucosal IgA, intrathecally produced dimeric IgA lacks the secretory component [47], which supports its intrathecal origin against translocation across epithelial cells and simply is not necessary to compare the milieus of CSF and mucosal surfaces. The diverse effects of dimeric secretory IgA on mucosal surfaces are well established: it safeguards against infections, acts as a barrier to toxins and other environmental threats on mucosal surfaces, and helps maintain immune balance by fostering a harmonious relationship with our microbiota [22]. In the MALT system, IgA responses are locally induced in a multitude of B cell follicles with germinal centers and are therefore T cell-dependent with affinity maturation (Figure 1). The resulting plasmablasts/cells exit via efferent lymphatics into the circulation and home back to the intestine through specific receptors (e.g., Alpha4beta7 integrin and MADCAM1), where they locally secrete IgA for translocation to the mucosal surface [49] (Figure 1). This local production and lack of availability of mucosal tissue is the main reason why it is so difficult to study the true nature of IgA immune responses; the predominantly monomeric IgA in blood differs significantly from dimeric mucosal IgA-mediated immunity.

In the CNS, IgA immunity is less a safeguard but rather required for adaptive immune defense in various settings. Investigating total intrathecal IgA production in the context of the so-called three-class (IgG/IgA/IgM) reaction hence has long been an integral part of the CSF laboratory diagnostics of neuroinflammation [50]. Accordingly, a dominant intrathecal IgA synthesis is diagnostically informative in neurotuberculosis [51], brain abscesses, or adrenoleukodystrophy [52]. Intrathecal IgA syntheses in the context of two- to three-class reactions are also observed in neuroborreliosis, various viral infections (mumps meningoencephalitis, tick-borne encephalitis, and VZV) [51,53], and opportunistic infections [52].

Though not routinely assessed, pathogen-specific intrathecal IgA responses have been further described as a common occurrence in three viral disease entities relevant to MS, i.e., herpes simplex encephalitis, VZV, and mumps meningitis [2,54]. The context of MS is important, as MS also appears to be associated with viral infections. Previous studies have detected a higher prevalence of viral DNA, mostly from different herpesviruses like human herpesvirus 6, and Epstein–Barr virus, as well as increased IgG viral titers in the CSF of MS patients compared to controls [55,56]. These viral infections have also been linked to an increased risk of disease exacerbations [57]. Growing evidence particularly supports the concept regarding the involvement of the Epstein–Barr virus in the pathogenesis of MS [58].

In summary, it has long been known that IgA is locally synthesized in CSF during neuroinfections, that neuroinfections can play a certain role in the immunopathogenesis and progression of MS, and that intrathecal IgA synthesis occurs both in neuroinfection and in MS. However, the reasons, mechanisms, and details remain unclear.

### 3.2. Role of the Gut–Brain Axis?

Whereas IgG and IgM constitute the main circulating humoral immunity routinely tested for diagnostic or vaccination status monitoring, it appears at first glimpse astonishing that mucosal immunity-associated IgA could play a significant role in the CNS. At a second glimpse, however, the gut–brain axis theory comes to mind, and in fact, its potential involvement in MS is a relatively new concept [59,60].

The central idea of this theory is that the gut microbiome and the brain communicate bidirectionally via the neuronal, immune, and endocrine systems and that the composition of the microbiome influences the development and function of the nervous system [61]. From the pathological viewpoint, individuals with MS exhibit an altered gut microbiome composition compared to healthy controls, characterized by reduced bacterial diversity and an increase in pro-inflammatory bacterial groups [59]. Remarkably, MS patients in relapse and remission also show significant differences in microbiome compositions, with the bacterial population in the remission group being more similar to that of healthy controls [59]. The proposed mechanism involves disrupting the delicate balance between the host immune system and the gut microbiota, which leads to increased inflammatory responses, altered T cell differentiation, and potential breaches in self-tolerance, ultimately resulting in systemic inflammation and autoimmune susceptibility [60].

Moreover, the breakdown of oral tolerance has long been implicated in the development of various animal and human autoimmune diseases including MS [62]. Rezende et al. recently highlighted the potential of harnessing oral tolerance as a therapeutic approach for autoimmune diseases [31]. The concept involves administering specific antigens orally to induce tolerance and suppress pathological immune responses. This approach has shown promise in experimental autoimmune encephalomyelitis (EAE), an animal model of MS. The growing understanding of the gut–brain axis and oral tolerance mechanisms offers new avenues for developing targeted, antigen-specific therapies for MS and other autoimmune diseases.

In line with the idea of oral tolerance being possibly linked to CNS immunity, Rojas et al. recently demonstrated that IgA-producing plasma cells and plasmablasts originating from the gut accumulate in the brain and spinal cord in MOG-induced EAE [63]. These gut-derived IgA+ plasma cells attenuated EAE in an Il-10-dependent manner, which could be enhanced by supplementing the already established microbiota with a singular IgA-promoting commensal microbe. Furthermore, mice lacking IgA-secreting cells exhibited more severe EAE symptoms, whereas mice overexpressing BAFF, which is a major survival factor for plasma cells, did not develop clinical signs of EAE at all. Intestinal IgA+ B cells hence might represent a reservoir of regulatory cells, which upon recruitment into inflamed tissues independent of receptor specificity act via immune regulatory Il-10. Such a scenario might further explain why CD20-targeting therapies are effective in MS whereas atacicept, which blocks the receptor of BAFF on plasma cells, leads to disease exacerbation [63].

Certainly, given the differences between mouse and human IgA systems, research in humans is needed to confirm whether IgA-producing B cells can infiltrate the brain and exert similar protective effects. In fact, the first corroborating evidence is already available with the work of Proebstel et al. and their finding that gut microbiota-specific IgA cells act as a systemic mediator between the gut and the CNS during active disease [46]. These IgA-producing cells were found to be specific for MS-associated taxa and upon trafficking to the inflamed CNS elicited a strong, compartmentalized enrichment in CSF of both IgA+ B cells and intrathecal IgA elevations during relapse, particularly of the IgA1 subclass.

Besides emphasizing intrathecal IgA as a marker for active MS, this research underscores the role of the gut–brain axis in CNS inflammation and gut-associated IgA+ B cells as targets for future treatment approaches. After all, it also provides a possible explanation for why dimeric IgA predominates in inflamed CSF.

## 4. Clinical Evidence Supporting a Prognostic Role of Intrathecal IgA Synthesis in MS

Given these experimental clues, intrathecal IgA elevations observed in MS may not be a trivial finding but have prognostic implications. To review the current state of clinical research, we conducted an advanced PubMed search using the string “IgA [Title/Abstract] AND cerebrospinal fluid [Title/Abstract] AND multiple sclerosis [MeSH Terms]”. The majority of publications dated back to the 1970s until the early 2000s and primarily focused on methodological aspects of detecting immunoglobulins in CSF in the context of neuroinflammation. Furthermore, we noticed a resurgence of interest in intrathecal IgA synthesis, which is probably based on the above-outlined deeper understanding of its biology due to technological and methodological advances.

Here, our focus was to retrieve clinical studies touching the context of the prognostic relevance of intrathecal IgA synthesis in MS, which we summarized in Table 1. Of the nine studies covering a period of more than three decades, only three investigated intrathecal IgA synthesis as the central research question [17,64,65], while in the remaining studies, intrathecal IgA synthesis was a secondary aspect.

The reviewed papers comprised five retrospective [17,45,64,66,67] and four prospective studies [9,12,65,68]. The patient cohorts of these studies were highly heterogeneous, with some focusing on relapsing–remitting forms [65], others on progressive forms [67], or both [9]. Three of the studies also included Clinically Isolated Syndrome (CIS) [12,17,66], whereas three other studies did not differentiate between disease courses at all [45,64,68]. Both treated and untreated patients were investigated; in one study, data from alive versus deceased patients were assessed as the main outcome [64]. Also, the methods used for detecting IgA varied widely, ranging from nephelometry [12,65,66,67] to ELISA [9,45,68] with the Reiber formula [12,17,45,67], the Auer and Hegen approach [66], or the IgA index [9,64,68], which are used for quantifying intrathecal IgA synthesis. Some studies also applied isoelectric focusing for the qualitative detection of intrathecal IgA synthesis [9,45,68]; different combinations of methods were frequent across studies (Table 1).

The substantial heterogeneity in study designs, cohort sizes, and composition, as well as the diversity in IgA detection techniques, makes it challenging to directly compare, summarize, and reconcile the partly conflicting results across studies.

Three studies suggested a positive prognostic value for intrathecal IgA synthesis [59,62,63]. These findings indicate that the presence of intrathecal IgA might be associated with a more favorable disease course or response to treatment.

Conversely, two studies proposed negative prognostic implications [9,60], with the results suggesting that intrathecal IgA synthesis could be linked to more aggressive disease progression or poorer outcomes.

Gasperi et al. reported no association between intrathecal IgA and disease worsening [12], while Rodriguez-Mogeda et al. did not detect intrathecal IgA synthesis in their MS cohort [45]. The results of these two studies could hence be categorized as neutral regarding the role of intrathecal IgA.

Two studies finally reached opposite conclusions regarding the relevance of intrathecal IgA detection in the CSF of MS patients, with Muñoz et al. [17] suggesting an underestimation and Berek et al. [66] indicating an overestimation.

All of these studies and the conflicting findings highlight a complex role and biology behind intrathecal IgA synthesis in MS, which is probably related to a specific subgroup of patients or disease states. Beyond that, methodological inconsistencies regarding detection and quantification as well as a lack of awareness of dimeric IgA forms in the inflamed CSF especially represent sources for these inconsistent results and emphasize the need for refined and standardized detection techniques.

## 5. Methodological Aspects of IgA Detection

Historically, the detection of IgA in the CSF of MS patients has been challenging due to its relatively low abundance, glycosylation patterns, and occurrence of IgA dimers in neuroinflammation [17].

Since then, the situation has not changed significantly. In current CSF routine laboratory practice, nephelometry is widely used for measuring IgA concentrations. IgA concentrations in both CSF and blood are evaluated via IgA Reibergrams or the IgA index for quantitative estimations of intrathecal production [50]. Both measures take into account the integrity of the blood–CSF barrier and distinguish between intrathecally synthesized IgA and IgA that has diffused from blood [50]. The downside of nephelometry, however, is that it lacks sensitivity to detect low levels of intrathecal IgA and that it is not informative regarding the presence of dimeric IgA forms.

Both issues were the basis of intensive methodological work about intrathecal IgA with the vast majority published in the previous century. This includes the above-described approach of Sindic et al., who applied ultracentrifugation in isokinetic sucrose gradients and a particle-counting immunoassay (PACIA) to demonstrate that intrathecally produced dimeric forms of IgA predominate in the inflamed CSF [47]. Such extensive methodological procedures, however, are not compatible with clinical routine.

A similar result was found for IgA-IEF. In 1994, Withold et al. developed an IgA-IEF protocol with which they found out that two-thirds of patients with neuroinflammatory disorders were negative in IgA Reibergrams but showed oligoclonal IgA [69]. Moreover, 17% of the patients had intrathecal IgA-OCB as the only evidence of intrathecal immunoglobulin synthesis. Despite its higher sensitivity compared to nephelometry, the IgA-IEF protocol was not adopted into clinical routine but fell prey to challenging sample pretreatment procedures. These procedures involved ultrafiltration for concentrating the CSF, the chemical reduction of dimeric IgA into monomeric IgA, specific gel preparations to avoid artificial precipitation of IgA, and appropriate detection antibodies specific for IgA, with their binding not compromised by IgA glycosylation patterns.

The rekindled interest in intrathecal IgA, however, led to new methodological efforts utilizing modern techniques and reagents. Accordingly, a high-sensitivity in-house ELISA assay capable of detecting IgA concentrations as low as 0.3125 ng/mL and an IgA IEF with a more straightforward protocol than that of Withold et al. with an improved sensitivity of the immunodetection were developed [17]. Applying these techniques, IgA-OCBs were detectable even in individuals with an intrathecal IgA concentration below the normal range. IgA-OCBs hence were observed at a higher prevalence as previously reported in neurological diseases and at higher frequencies as previously described in MS [17]. Of note, authors reported IgA-OCB pattern III (identical OCBs detectable in CSF and sera with additional bands in the CSF) predominating in MS and a considerable percentage of MS patients showing pattern IV (identical bands in both CSF and sera). This indicates humoral IgA-related B cell activation in the periphery and emphasizes the need for broadly established methods allowing tracking of intrathecal IgA synthesis.

## 6. Conclusions and Future Perspective

The importance of reassessing intrathecal IgA synthesis in MS is evident, challenging the prevailing belief that it is rare and irrelevant. This reassessment requires addressing methodological challenges such as low abundance, glycosylation, and dimerization of IgA, as well as developing standardized, highly sensitive detection assays. A window of opportunity may evolve from translating novel assays from infectiology into neuroimmunology. The necessity to track COVID-19 infections led to the development of a multitude of IgA-based immunoassays, including immunological methods for detecting dimeric IgA in patients’ sera as a marker for recent infection [70].

Understanding the true biology of intrathecal IgA synthesis necessitates investigating the cellular and immune mediator context, including IgA+ B cells, IgA-secreting plasmablasts/cells, and associated cytokine profiles in CSF.

While intrathecal IgA synthesis is detectable in MS patients at varying frequencies, its clinical significance remains unclear. We here summarized accumulating evidence supporting its relevance. Particularly, experimental models showing that IgA-specific plasma cells can migrate from the gut to the brain highlight a potential neuroprotective role by producing the anti-inflammatory cytokine IL-10. These findings from mouse studies have been further extended to MS patients, indicating that gut microbiota-specific IgA-producing cells function as systemic mediators in MS. In the same study, elevated levels of IgA in CSF were associated with relapses and acute inflammation. A compelling explanation for this phenomenon is the recruitment of gut-derived IgA-producing B cells to the inflamed CNS, where they exert regulatory and anti-inflammatory functions. Moreover, the observed predominance of pattern 3 IgA-OCBs might indicate a systemic tolerance pathway extending to the CNS and implicating a better prognosis in a subset of MS patients. The presence of a unique immune regulatory B cell population may promote more favorable disease characteristics.

A critical future research task would be to investigate this concept using modern, robust multicenter study designs. Identifying a link to oral and systemic tolerance via the gut microbiome would provide valuable knowledge for developing future therapeutic strategies in the treatment of MS.

An integrated approach involving the detection of intrathecal IgA+ B cells, plasma cells, IgA subclasses, dimerization, cytokine environment, and regulatory T cell subpopulations will be essential to determine whether intrathecal IgA synthesis is an epiphenomenon or a surrogate marker of a pro- or anti-inflammatory environment. Further research is crucial to clarify the role of IgA and IgA-associated immunity in MS pathogenesis and progression and to establish its place in diagnosis, prognosis, and potentially even treatment approaches.

## Figures and Tables

**Table 1:** Overview of clinical studies exploring prognostic implications of intrathecal IgAproduction in multiple sclerosis.

Authors, Year, Ref.	Study Type	MS Patients (n)	Intrathecal IgA Synthesis	Findings	Role of IgA
Rodriguez-Mogeda C, 2024 [42]	retrospective	80 MS	ELISA, Index and Reiber formula **/histology	No increased IgA synthesis in MS compared to controls; no association to axon loss; fewer IgA+ than IgG+ B cells in SPMS meninges; no association of intrathecal IgA production and presence of IgA+ B cells	Undetermined
Muñoz et al., 2022 [17]	retrospective	151 (CIS, RRMS, PMS)	IgA-IEF; ELISA, Reiber formula **	A highly sensitive IEF-based assay reveals intrathecal IgA synthesis more abundant particularly in CIS patients; patterns III and IV predominate demonstrating presence of peripheral activated B cells	Intrathecal IgA synthesis in MS is underestimated
Berek et al., 2021 [61]	retrospective	541 (CIS, RRMS, SPMS, PPMS)	Nephelometry; Reiber formula **, Auer and Hegen formula ***	Applying their novel reference, limits intrathecal IgA synthesis was less frequent	Intrathecal IgA synthesis in MS is overestimated
Kroth et al., 2019 [60]	prospective	71 RRMS (11% untreated)	Nephelometry; intrathecal Ig synthesis not determined	Baseline QIgA values correlated with the rate of cortical atrophy and higher CSF IgA levels were mirrored by increased regional grey matter loss after a one-year follow-up	Negative prognostic value
Gasperi et al., 2019 [12]	prospective	673 (RRMS, CIS)	Nephelometry/turbidimetry, Reiber formula **	No association between intrathecal IgA synthesis and risk of/time to EDSS worsening	No negative prognostic value
Abdelhak et al., 2017 [62]	retrospective	254 PPMS	Nephelometry, Reiber formula **	A moderate negative correlation was found between intrathecal IgA synthesis and the yearly progression rate	Possible positive prognostic value
Vrethem et al., 2024 [59]	retrospective	71 MS (alive vs. dead)	Nephelometry, IgA Index/extended IgA index *	Deceased patients had lower intrathecal IgA synthesis; no association with disability score	Possible positive prognostic value
Sellebjerg et al., 1998 [63]	prospective	49 MS	ELISA, IgA index	Intrathecal IgA synthesis inversely correlated with progression	Positive prognostic value
Lolli et al., 1989 [9]	prospective	61 (RRMS, SPMS)	ELISA, IgA index	Patients with chronic progressive MS had higher frequency of intrathecal IgA synthesis	Possible negative prognostic value

* Öhman at al. Clin Chim Acta 1989; 181: 265 /72. ** Reiber et al. J Neurol Sci 2001; 184: 101–22. *** Auer et al. Eur J Neurol 2016; 23 (4): 713-21. Abbreviations: CIS, clinically isolated syndrome; ELISA, Enzyme Linked Immunosorbent Assay; IEF, isoelectric focusing; MS, multiple sclerosis; PPMS, primary progressive MS; RRMS, relapsing-remitting MS; SPMS, secondary progressive MS.

## Data Availability

No new data were created.

## References

[1] Reiber H. (1998). Cerebrospinal fluid—Physiology, analysis and interpretation of protein patterns for diagnosis of neurological diseases. Mult. Scler. J..

[2] Reiber H., Ungefehr S., Jacobi C. (1998). The intrathecal, polyspecific and oligoclonal immune response in multiple sclerosis. Mult. Scler. J..

[3] Freedman M.S., Thompson E.J., Deisenhammer F., Giovannoni G., Grimsley G., Keir G., Öhman S., Racke M.K., Sharief M., Sindic C.J.M. (2005). Recommended standard of cerebrospinal fluid analysis in the diagnosis of multiple sclerosis: A consensus statement. Arch. Neurol..

[4] Hauser S.L., Waubant E., Arnold D.L., Vollmer T., Antel J., Fox R.J., Bar-Or A., Panzara M., Sarkar N., Agarwal S. (2008). B-cell depletion with rituximab in relapsing-remitting multiple sclerosis. N. Engl. J. Med..

[5] Hauser S.L. (2015). The Charcot Lecture|beating MS: A story of B cells, with twists and turns. Mult. Scler. J..

[6] Hauser S.L., Bar-Or A., Comi G., Giovannoni G., Hartung H.P., Hemmer B., Lublin F., Montalban X., Rammohan K.W., Selmaj K. (2017). Ocrelizumab versus Interferon Beta-1a in Relapsing Multiple Sclerosis. N. Engl. J. Med..

[7] Comi G., Bar-Or A., Lassmann H., Uccelli A., Hartung H.P., Montalban X., Sorensen P.S., Hohlfeld R., Hauser S.L. (2021). Role of B Cells in Multiple Sclerosis and Related Disorders. Ann. Neurol..

[8] Henriksson A., Kam-Hansen S., Link H. (1985). IgM, IgA and IgG producing cells in cerebrospinal fluid and peripheral blood in multiple sclerosis. Clin. Exp. Immunol..

[9] Lolli F., Halawa I., Link H. (1989). Intrathecal synthesis of IgG, IgA, IgM and IgD in untreated multiple sclerosis and controls. Acta Neurol. Scand..

[10] Jin H., Lu Q., Gao F., Hao H. (2023). Application of oligoclonal bands and other cerebrospinal fluid variables in multiple sclerosis and other neuroimmunological diseases: A narrative review. Ann. Transl. Med..

[11] Dobson R., Ramagopalan S., Davis A., Giovannoni G. (2013). Cerebrospinal fluid oligoclonal bands in multiple sclerosis and clinically isolated syndromes: A meta-analysis of prevalence, prognosis and effect of latitude. J. Neurol. Neurosurg. Psychiatry.

[12] Gasperi C., Salmen A., Antony G., Bayas A., Heesen C., Kumpfel T., Linker R.A., Paul F., Stangel M., Tackenberg B. (2019). Association of Intrathecal Immunoglobulin G Synthesis With Disability Worsening in Multiple Sclerosis. JAMA Neurol..

[13] Villar L.M., Masjuan J., Gonzalez-Porque P., Plaza J., Sadaba M.C., Roldan E., Bootello A., Alvarez-Cermeno J.C. (2002). Intrathecal IgM synthesis predicts the onset of new relapses and a worse disease course in MS. Neurology.

[14] Oechtering J., Schaedelin S., Benkert P., Muller S., Achtnichts L., Vehoff J., Disanto G., Findling O., Fischer-Barnicol B., Orleth A. (2021). Intrathecal Immunoglobulin M Synthesis is an Independent Biomarker for Higher Disease Activity and Severity in Multiple Sclerosis. Ann. Neurol..

[15] Mailand M.T., Frederiksen J.L. (2020). Intrathecal IgM as a Prognostic Marker in Multiple Sclerosis. Mol. Diagn. Ther..

[16] Huss A., Abdelhak A., Halbgebauer S., Mayer B., Senel M., Otto M., Tumani H. (2018). Intrathecal immunoglobulin M production: A promising high-risk marker in clinically isolated syndrome patients. Ann. Neurol..

[17] Munoz U., Sebal C., Escudero E., Garcia Sanchez M.I., Urcelay E., Jayo A., Arroyo R., Garcia-Martinez M.A., Alvarez-Lafuente R., Sadaba M.C. (2022). High prevalence of intrathecal IgA synthesis in multiple sclerosis patients. Sci. Rep..

[18] Gommerman J.L., Rojas O.L., Fritz J.H. (2014). Re-thinking the functions of IgA(+) plasma cells. Gut Microbes.

[19] Cyster J.G., Allen C.D.C. (2019). B Cell Responses: Cell Interaction Dynamics and Decisions. Cell.

[20] Nutt S.L., Hodgkin P.D., Tarlinton D.M., Corcoran L.M. (2015). The generation of antibody-secreting plasma cells. Nat. Rev. Immunol..

[21] Li Z., Woo C.J., Iglesias-Ussel M.D., Ronai D., Scharff M.D. (2004). The generation of antibody diversity through somatic hypermutation and class switch recombination. Genes Dev..

[22] Seikrit C., Pabst O. (2021). The immune landscape of IgA induction in the gut. Semin. Immunopathol..

[23] Mestecky J., Russell M.W., Jackson S., Brown T.A. (1986). The human IgA system: A reassessment. Clin. Immunol. Immunopathol..

[24] Kerr M.A. (1990). The structure and function of human IgA. Biochem. J..

[25] de Sousa-Pereira P., Woof J.M. (2019). IgA: Structure, Function, and Developability. Antibodies.

[26] Brandtzaeg P., Johansen F.E. (2005). Mucosal B cells: Phenotypic characteristics, transcriptional regulation, and homing properties. Immunol. Rev..

[27] Pabst O. (2012). New concepts in the generation and functions of IgA. Nat. Rev. Immunol..

[28] Steffen U., Koeleman C.A., Sokolova M.V., Bang H., Kleyer A., Rech J., Unterweger H., Schicht M., Garreis F., Hahn J. (2020). IgA subclasses have different effector functions associated with distinct glycosylation profiles. Nat. Commun..

[29] Pier J., Liu E.G., Eisenbarth S., Jarvinen K.M. (2021). The role of immunoglobulin A in oral tolerance and food allergy. Ann. Allergy Asthma Immunol..

[30] Brandtzaeg P. (2010). Food allergy: Separating the science from the mythology. Nat. Rev. Gastroenterol. Hepatol..

[31] Rezende R.M., Weiner H.L. (2022). Oral tolerance: An updated review. Immunol. Lett..

[32] Engelhardt B., Ransohoff R.M. (2012). Capture, crawl, cross: The T cell code to breach the blood-brain barriers. Trends Immunol..

[33] Diaz-Coranguez M., Ramos C., Antonetti D.A. (2017). The inner blood-retinal barrier: Cellular basis and development. Vision Res..

[34] Millan Solano M.V., Salinas Lara C., Sanchez-Garibay C., Soto-Rojas L.O., Escobedo-Avila I., Tena-Suck M.L., Ortiz-Butron R., Choreno-Parra J.A., Romero-Lopez J.P., Melendez Camargo M.E. (2023). Effect of Systemic Inflammation in the CNS: A Silent History of Neuronal Damage. Int. J. Mol. Sci..

[35] Pollok K., Mothes R., Ulbricht C., Liebheit A., Gerken J.D., Uhlmann S., Paul F., Niesner R., Radbruch H., Hauser A.E. (2017). The chronically inflamed central nervous system provides niches for long-lived plasma cells. Acta Neuropathol. Commun..

[36] Bogers L., Engelenburg H.J., Janssen M., Unger P.A., Melief M.J., Wierenga-Wolf A.F., Hsiao C.C., Mason M.R.J., Hamann J., van Langelaar J. (2023). Selective emergence of antibody-secreting cells in the multiple sclerosis brain. EBioMedicine.

[37] Corcione A., Aloisi F., Serafini B., Capello E., Mancardi G.L., Pistoia V., Uccelli A. (2005). B-cell differentiation in the CNS of patients with multiple sclerosis. Autoimmun. Rev..

[38] Kee R., Naughton M., McDonnell G.V., Howell O.W., Fitzgerald D.C. (2022). A Review of Compartmentalised Inflammation and Tertiary Lymphoid Structures in the Pathophysiology of Multiple Sclerosis. Biomedicines.

[39] Mitsdoerffer M., Peters A. (2016). Tertiary Lymphoid Organs in Central Nervous System Autoimmunity. Front. Immunol..

[40] Howell O.W., Reeves C.A., Nicholas R., Carassiti D., Radotra B., Gentleman S.M., Serafini B., Aloisi F., Roncaroli F., Magliozzi R. (2011). Meningeal inflammation is widespread and linked to cortical pathology in multiple sclerosis. Brain.

[41] Magliozzi R., Howell O., Vora A., Serafini B., Nicholas R., Puopolo M., Reynolds R., Aloisi F. (2007). Meningeal B-cell follicles in secondary progressive multiple sclerosis associate with early onset of disease and severe cortical pathology. Brain.

[42] Magliozzi R., Howell O.W., Calabrese M., Reynolds R. (2023). Meningeal inflammation as a driver of cortical grey matter pathology and clinical progression in multiple sclerosis. Nat. Rev. Neurol..

[43] Stern J.N., Yaari G., Vander Heiden J.A., Church G., Donahue W.F., Hintzen R.Q., Huttner A.J., Laman J.D., Nagra R.M., Nylander A. (2014). B cells populating the multiple sclerosis brain mature in the draining cervical lymph nodes. Sci. Transl. Med..

[44] Zhang Y., Da R.R., Hilgenberg L.G., Tourtellotte W.W., Sobel R.A., Smith M.A., Olek M., Nagra R., Sudhir G., van den Noort S. (2005). Clonal expansion of IgA-positive plasma cells and axon-reactive antibodies in MS lesions. J. Neuroimmunol..

[45] Rodriguez-Mogeda C., van Gool M.M., van der Mast R., Nijland R., Keasberry Z., van de Bovekamp L., van Delft M.A., Picon C., Reynolds R., Killestein J. (2024). Intrathecal IgG and IgM synthesis correlates with neurodegeneration markers and corresponds to meningeal B cell presence in MS. Sci. Rep..

[46] Probstel A.K., Zhou X., Baumann R., Wischnewski S., Kutza M., Rojas O.L., Sellrie K., Bischof A., Kim K., Ramesh A. (2020). Gut microbiota-specific IgA(+) B cells traffic to the CNS in active multiple sclerosis. Sci. Immunol..

[47] Sindic C.J., Delacroix D.L., Vaerman J.P., Laterre E.C., Masson P.L. (1984). Study of IgA in the cerebrospinal fluid of neurological patients with special reference to size, subclass and local production. J. Neuroimmunol..

[48] Hu W.C. (2020). A Framework of All Discovered Immunological Pathways and Their Roles for Four Specific Types of Pathogens and Hypersensitivities. Front. Immunol..

[49] Wang C., McDonough J.S., McDonald K.G., Huang C., Newberry R.D. (2008). Alpha4beta7/MAdCAM-1 interactions play an essential role in transitioning cryptopatches into isolated lymphoid follicles and a nonessential role in cryptopatch formation. J. Immunol..

[50] Tumani H., Petereit H.F., Gerritzen A., Gross C.C., Huss A., Isenmann S., Jesse S., Khalil M., Lewczuk P., Lewerenz J. (2020). S1 guidelines “lumbar puncture and cerebrospinal fluid analysis” (abridged and translated version). Neurol. Res. Pract..

[51] Felgenhauer K., Schadlich H.J. (1987). The compartmental IgM and IgA response within the central nervous system. J. Neurol. Sci..

[52] Reiber H., Peter J.B. (2001). Cerebrospinal fluid analysis: Disease-related data patterns and evaluation programs. J. Neurol. Sci..

[53] Kaiser R. (1999). The clinical and epidemiological profile of tick-borne encephalitis in southern Germany 1994–98: A prospective study of 656 patients. Brain.

[54] Roberg M., Forsberg P., Tegnell A., Ekerfeldt K. (1995). Intrathecal production of specific IgA antibodies in CNS infections. J. Neurol..

[55] Tzartos J.S., Khan G., Vossenkamper A., Cruz-Sadaba M., Lonardi S., Sefia E., Meager A., Elia A., Middeldorp J.M., Clemens M. (2012). Association of innate immune activation with latent Epstein-Barr virus in active MS lesions. Neurology.

[56] Alvarez-Lafuente R., Garcia-Montojo M., De Las Heras V., Dominguez-Mozo M.I., Bartolome M., Benito-Martin M.S., Arroyo R. (2008). Herpesviruses and human endogenous retroviral sequences in the cerebrospinal fluid of multiple sclerosis patients. Mult. Scler..

[57] Buljevac D., Flach H.Z., Hop W.C., Hijdra D., Laman J.D., Savelkoul H.F., van Der Meche F.G., van Doorn P.A., Hintzen R.Q. (2002). Prospective study on the relationship between infections and multiple sclerosis exacerbations. Brain.

[58] Bjornevik K., Cortese M., Healy B.C., Kuhle J., Mina M.J., Leng Y., Elledge S.J., Niebuhr D.W., Scher A.I., Munger K.L. (2022). Longitudinal analysis reveals high prevalence of Epstein-Barr virus associated with multiple sclerosis. Science.

[59] Chen J., Chia N., Kalari K.R., Yao J.Z., Novotna M., Paz Soldan M.M., Luckey D.H., Marietta E.V., Jeraldo P.R., Chen X. (2016). Multiple sclerosis patients have a distinct gut microbiota compared to healthy controls. Sci. Rep..

[60] Brown E.M., Kenny D.J., Xavier R.J. (2019). Gut Microbiota Regulation of T Cells During Inflammation and Autoimmunity. Annu. Rev. Immunol..

[61] Ullah H., Arbab S., Tian Y., Liu C.Q., Chen Y., Qijie L., Khan M.I.U., Hassan I.U., Li K. (2023). The gut microbiota-brain axis in neurological disorder. Front. Neurosci..

[62] Weiner H.L., Friedman A., Miller A., Khoury S.J., al-Sabbagh A., Santos L., Sayegh M., Nussenblatt R.B., Trentham D.E., Hafler D.A. (1994). Oral tolerance: Immunologic mechanisms and treatment of animal and human organ-specific autoimmune diseases by oral administration of autoantigens. Annu. Rev. Immunol..

[63] Rojas O.L., Probstel A.K., Porfilio E.A., Wang A.A., Charabati M., Sun T., Lee D.S.W., Galicia G., Ramaglia V., Ward L.A. (2019). Recirculating Intestinal IgA-Producing Cells Regulate Neuroinflammation via IL-10. Cell.

[64] Vrethem M., Fernlund I., Ernerudh J., Ohman S. (2004). Prognostic value of cerebrospinal fluid IgA and IgG in multiple sclerosis. Mult Scler..

[65] Kroth J., Ciolac D., Fleischer V., Koirala N., Kramer J., Muthuraman M., Luessi F., Bittner S., Gonzalez-Escamilla G., Zipp F. (2019). Increased cerebrospinal fluid albumin and immunoglobulin A fractions forecast cortical atrophy and longitudinal functional deterioration in relapsing-remitting multiple sclerosis. Mult. Scler..

[66] Berek K., Bsteh G., Auer M., Di Pauli F., Zinganell A., Berger T., Deisenhammer F., Hegen H. (2021). Cerebrospinal Fluid Findings in 541 Patients With Clinically Isolated Syndrome and Multiple Sclerosis: A Monocentric Study. Front. Immunol..

[67] Abdelhak A., Hottenrott T., Mayer C., Hintereder G., Zettl U.K., Stich O., Tumani H. (2017). CSF profile in primary progressive multiple sclerosis: Re-exploring the basics. PLoS ONE.

[68] Sellebjerg F., Christiansen M., Nielsen P.M., Frederiksen J.L. (1998). Cerebrospinal fluid measures of disease activity in patients with multiple sclerosis. Mult. Scler..

[69] Withold W., Wick M., Fateh-Moghadam A., Einhaupl K. (1994). Detection of oligoclonal IgA in cerebrospinal fluid samples by an isoelectric focusing procedure. J. Neurol..

[70] Drummer H.E., Van H., Klock E., Zheng S., Wei Z., Boo I., Center R.J., Li F., Bhat P., Ffrench R. (2021). Dimeric IgA is a specific biomarker of recent SARS-CoV-2 infection. medRxiv.

